# Association of DNA methylation with age, gender, and smoking in an Arab population

**DOI:** 10.1186/s13148-014-0040-6

**Published:** 2015-01-22

**Authors:** Shaza B Zaghlool, Mashael Al-Shafai, Wadha A Al Muftah, Pankaj Kumar, Mario Falchi, Karsten Suhre

**Affiliations:** Bioinformatics Core, Weill Cornell Medical College in Qatar, Education City, PO Box 24144, Doha, Qatar; Computer Engineering Department, Virginia Tech, Blacksburg, VA 24060 USA; Department of Genomics of Common Disease, Imperial College London, London, UK; Research Division, Qatar Science Leadership Program, Qatar Foundation, Doha, Qatar; Helmholtz Zentrum München, Germany, Research Center for Environmental Health, 85764 Neuherberg, Germany

**Keywords:** DNA methylation, Age, Gender, Smoking, Association study, Epigenetics

## Abstract

**Background:**

Modification of DNA by methylation of cytosines at CpG dinucleotides is a widespread phenomenon that leads to changes in gene expression, thereby influencing and regulating many biological processes. Recent technical advances in the genome-wide determination of single-base DNA-methylation enabled epigenome-wide association studies (EWASs). Early EWASs established robust associations between age and gender with the degree of CpG methylation at specific sites. Other studies uncovered associations with cigarette smoking. However, so far these studies were mainly conducted in Caucasians, raising the question of whether these findings can also be extrapolated to other populations.

**Results:**

Here, we present an EWAS with age, gender, and smoking status in a family study of 123 individuals of Arab descent. We determined DNA methylation at over 450,000 CpG sites using the Illumina Infinium HumanMethylation450 BeadChip, applied state-of-the-art data processing protocols, including correction for blood cell type heterogeneity and hidden confounders, and eliminated probes containing SNPs at the targeted CpG site using 40× whole-genome sequencing data. Using this approach, we could replicate the leading published EWAS associations with age, gender and smoking, and recovered hallmarks of gender-specific epigenetic changes. Interestingly, we could even replicate the recently reported precise prediction of chronological age based on the methylation of only a few selected CpG sites.

**Conclusion:**

Our study supports the view that when applied with state-of-the art protocols to account for all potential confounders, DNA methylation arrays represent powerful tools for EWAS with more complex phenotypes that can also be successfully applied to non-Caucasian populations.

**Electronic supplementary material:**

The online version of this article (doi:10.1186/s13148-014-0040-6) contains supplementary material, which is available to authorized users.

## Background

DNA methylation is a chemical process where a methyl group is attached to the DNA at a CpG site. A CpG site is a DNA region where a cytosine nucleotide is found next to a guanine in the genome sequence, connected by a phosphate group. This process is catalyzed by a number of DNA methyltransferase enzymes [1]. DNA methylation is thought to be mostly established at an early embryonic state and then stably propagated through mitosis. However, genes are known to be dynamically regulated by a variety of factors including modifications of DNA and histones. During mitosis, maintenance mechanisms ensure that symmetrically methylated CpGs are reestablished in both daughter cells. Most genomic methylation patterns are thought to remain largely unchanged across tissues and throughout life, changing only in localized settings under specific conditions as cellular processes are activated or shut down [2]. For example, during mammalian development, most CpGs remain methylated while CpG islands located in the promoters of housekeeping genes are hypomethylated [3]. However, recent studies suggest that changes in DNA methylation, possibly in reaction to changes in lifestyle and environmental factors, can result in both global and localized epigenetic changes [4-6].

DNA methylation is one of the most commonly studied epigenetic regulation mechanisms and is involved in the regulation of many biological processes through the regulation of gene expression. One of the main roles of epigenetic modifications through DNA methylation is to control gene transcription in response to external and internal stimuli by targeting specific regulatory DNA bases, such as promoter and enhancer regions [7-9]. Another important biological process that is controlled by DNA methylation is the maintenance of gene imprinting [10]. This is a process where CpG sites are differentially methylated depending on their parental origins, which are not equivalent for paternal and maternal genomes. DNA methylation has also been known to be involved in phenomena like X-chromosome inactivation where one of the two copies of the X chromosome present in females is inactivated [11]. The global DNA methylation landscape is quite stable throughout the genome in mammalian embryos [12]. In human preimplantation embryos, however, the paternal genome is dynamically reprogrammed through temporary predominant demethylation of the majority of CpGs that is later reversed [12]. It was shown that the majority of this genome-wide demethylation is complete at the two-cell stage and that the demethylation process is much faster in males than in females [13].

A single CpG site in a single cell can be either methylated or unmethylated (binary mark). However, since there are two copies of each chromosome in every cell, between which most methods to determine CpG methylation cannot distinguish, any specific CpG site in a single cell can be found in a methylated, a hemi-methylated, or an unmethylated state. Moreover, most methylation measurements are not done on DNA from a single cell but are determined as an average methylation level of an ensemble of cells, including potentially even different cell types. Thus, the numeric value of the methylation state of a given CpG site is generally represented as the fraction of sites that are methylated in any given sample, often referred to as the B-value.

Recent advances in experimental techniques, such as the development of reduced representation bisulfite sequencing and of array-based DNA methylation assays, allow to determine DNA methylation on a genome-wide scale in hundreds or even thousands of individuals [14]. Using an epigenome-wide association study (EWAS) approach, differentially methylated DNA regions that are associated with phenotypes of medical interest can then be identified. EWAS with complex disorders, such as obesity [15-17], diabetes risk factors [18], rheumatoid arthritis [19], and metabolic traits [20] have already been reported. Differential methylation has also been linked to numerous other phenotypes, including smoking [21-24], age [25-30], and gender [31-33]. DNA methylation has even been shown to be a precise predictor of chronological age [34,35]. Although it is likely that many associations between DNA methylation and phenotype represent a general phenomenon, population-specific differences may exist and need to be addressed. In this paper, we are interested in identifying and replicating DNA methylation associations with age, gender, and smoking in a new dataset with individuals of Arab ethnicity.

As the technology used in the quantification of epigenetic modification is advancing in terms of throughput and coverage, the use of state-of-the-art data processing methodology is essential. This methodology should take into account the various biological, environmental, and technological factors involved. In this paper, we combine a series of preprocessing steps to ensure that potential sources of experimental bias are addressed in the proper context, and that results are independent of systematic technical variations. Biological confounders, such as differences in cell type composition, need to be accounted for in such analyses. Experimental artifacts, both, known systematic errors and confounders of unknown origin, can also contribute to this problem and require adequate preprocessing before subsequent analysis. A preprocessing pipeline was therefore applied to a dataset that we collected from a population of consanguineous families of Arab origin, prior to performing a comprehensive genome-wide association study of epigenetics with the phenotypes age, gender, and smoking status.

## Results

### Study population and data collection

The study’s subjects consist of Qataris who are natives of the Arabian Peninsula, a region that is part of the Middle East. Most of the population descended from several migratory tribes that came to Qatar in the eighteenth century to escape tough conditions of the nearby areas. The ancestral background of the Qataris is a combination of mostly Bedouins, Persians or South Asian mixture, and African-derived Qataris which form a quite broad genomic makeup. The Qatari population is characterized by a large number of consanguineous families sharing the same ancestor, often between first or second cousins. The study population was selected from two different datasets, which were collected initially for genetic family-based studies on type 2 diabetes and obesity. Initial contacts with probands were made through their regular follow-up visits to the Qatar Diabetes Association (QDA), a secondary health-care center that provides patients care, education, and support. Probands were asked to complete a patients’ information sheet to include full name, age, date of birth, gender, address, telephone number, and number of family members. Home visits were then arranged 2–3 days before the visit to collect blood samples and phenotypic information from all family members.

One hundred and twenty-three adult individuals of Qatari nationality, including 72 females with mean age 39 ± 16.9 and 51 males with mean age 36.3 ± 17.2, were investigated. Phenotype measurements included age, gender, body mass index (BMI), and smoking status (self-reported). The mean body mass index of the females was 28.3 ± 6.2 kg/m^2^ and of the males was 29.2 ± 7.2 kg/m^2^. The dataset comprised 13 smokers, all of whom were males, and 108 non-smokers. The dataset consisted of 16 families of various sizes having a variety of complex pedigree structures. All the subjects also responded to general health and lifestyle questionnaires.

We obtained DNA from whole blood samples and submitted them both to genome-wide methylation array analysis and to whole-genome sequencing, all performed at Illumina Inc. (San Diego CA, USA) on a fee-for-service basis. Methylation measurements were done using the Illumina Infinium HumanMethylation450 BeadChip Kit array (referred to as 450 K array in the following). Whole-genome sequencing (WGS) (40× coverage) was performed on the Illumina Hiseq 2500 platform. All measurements were done following standard Illumina protocols and procedures (see Methods). In total, we obtained methylation data for 485,577 sites and 14,595,042 genetic variants, called in at least one individual at a quality cut-off of q20.

### Data processing

Based on the comparison of six different analysis pipelines [36], the Lumi:QN + BMIQ pipeline was shown to be the most optimally designed for preprocessing of Illumina 450 K array data and was therefore applied here (see Methods). Briefly, the methylation data was first cleaned using a series of filtering steps. This included the exclusion of non-CpG sites and low quality sites and samples. Based on the WGS data, we further set all individual CpG sites containing a SNP in the probe locus (+/−110 nucleotides of the CpG site) to missing. This resulted in a total of 468,375 sites and 123 samples for the analysis (no samples were excluded). The effects of color bias adjustment and quantile normalization are shown in Additional file 1: Figure S1. Type 2 probes constitute 72% of the probes (two different color channels) while the remainder (28%) are type 1 in which both signals are obtained using the same color channel. BMIQ normalization is used so that type 1 peaks are matched to the normalized type 2 peaks at the methylation extremes. The effect of this normalization on our dataset can be seen as an example in Additional file 2: Figure S2 where the difference in peaks can be seen at the fully methylated end in the form of a bimodal peak (a) whereas this effect is removed after normalization (b).

Since DNA was collected from whole blood, white blood cell heterogeneity needs to be corrected for. The method described by Houseman et al. [37] allows the estimation of the cell type composition from whole-genome methylation data. The fraction of six cell types, namely monocytes, granulocytes, NK cells, B cells, CD8^+^-T cells, and CD4^+^-T cells, were determined for each subject using the methylSpectrum software. The resulting distribution plots of the white blood cell coefficients for our dataset are presented in Additional file 3: Figure S3.

In order to capture any remaining potential confounders in the methylation data, principal component analysis (PCA), as described and implemented in the software package MethylPCA [38], was used. The first ten principal components (PCs) were computed for each subject after first regressing out the known covariates age, gender, BMI, smoking state, and the blood composition coefficients. These ten components captured over 99% of the total variance that was still present in the data. Additional file 4: Figure S4 shows the estimated percentage of the variance that was captured by each of the individual PCs. In order to determine the association between CpG methylation and the different phenotypes of interest, we used linear regression models while accounting for the various covariates, white blood cell composition and principal components (see next section).

### Smoking-related differential methylation

**Figure 1:**
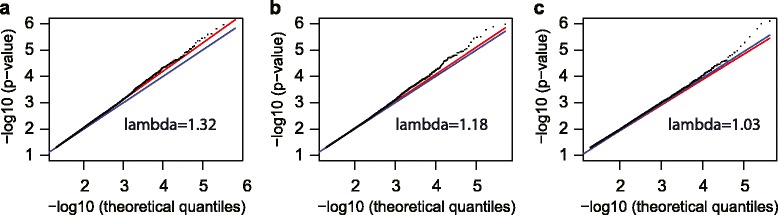
**Q-Q plots for the association between smoking and each methylation site: a) before performing PCA (including age, gender, BMI in the model), inflation coefficient = 1.32; b) before performing PCA (including age, gender, BMI and cell coefficients for monocytes and granulocytes), inflation coefficient = 1.18; and c) after performing PCA and (including age, gender, BMI, monocytes and granulocytes, PC1), inflation coefficient = 1.03.**

To identify the relevant covariates to include into the final model, we evaluated the effect of including the estimated white blood cell coefficients and the PCs on the *p* value distribution. Details are described in the Methods section. Figure 1 shows the association tests for the smoking phenotype in the form of Q-Q plots and the inflation factor lambda values for different linear models [39]. The cell composition coefficients were added to the model in Figure 1b, which reduced the inflation factor from using the base model (Figure 1a) from 1.32 to 1.18, verifying the confounding effect of cell-type variability. The inflation factor was further reduced between Figure 1b and 1c from 1.18 to 1.03 by adding principal component 1 (PC1) as additional covariate, suggesting that there were still unmeasured sources of variability other than cell composition present in our dataset that needed to be accounted for.

As can be seen from the Q-Q plot in Figure 1c, associations of methylation with respect to tobacco smoking deviate from the null at low *p* values. The top three hits were observed at methylation sites within the *AHRR* gene (cg0575921, cg26703534, cg14647125; *p* values between 7.47 × 10^−7^ and 3.88 × 10^−5^), thus replicating previous findings reported in other populations [21-24]. Table 1 shows this data along with other previously reported smoking-associated genes that did not rank highest in our data but still showed an association with smoking in our dataset at a false discovery rate of FDR <0.05.

**Table 1:** **CpG sites associated with smoking**

**CpG**	**Gene**	***p*** **value**	**Reference**
cg05575921	AHRR	7.47 × 10^−7^	[21,23,24]
cg26703534	AHRR	7.20 × 10^−6^	[21]
cg14647125	AHRR	3.88 × 10^−5^	
cg03636183	F2RL3	5.13 × 10^−4^	[21,22]
cg19859270	GPR1	1.85 × 10^−3^	[22]
cg21161138	AHRR	2.08 × 10^−3^	[21,23]
cg14817490	AHRR	3.68 × 10^−3^	[24]
cg10399789	GFI1	5.80 × 10^−3^	[21]

Although we have only a limited number of smokers in our dataset, we believe that since we are not claiming that our findings are a new discovery but merely replicating a specific CpG locus that was previously reported in a number of publications, statistical power is not as essential as it would be when claiming new discoveries. Actually, the fact that cg05575921 in AHRR turned up as the most significant hit in our data (as in previous studies), regardless of the limited number of subjects, suggests that the signal is quite strong. We performed a Wilcoxon rank test as a sensitivity analysis to show that our associations are quite robust even for this small number of subjects (i.e. accounting for potential effects of violation of normality). Since our *p* values relative to the rank test *p* values remained nominally significant when using a statistically less powerful but more robust test (data not shown), we can confirm that our small sample size and the deviation from normality do not alter the main findings/conclusions presented in this paper.

### Gender-related differential methylation

We next conducted an EWAS to identify specific sites that exhibit a gender-specific pattern. A total of 9,630 CpG sites showed genome-wide significant association with gender using a conservative Bonferroni threshold of 1.07 × 10^−7^ (0.05/468,375). Out of these 9,630 significant CpG sites, 7,155 were mapped to genes according to the Illumina Human Methylation 450 K annotation database that was assembled using data from public repositories (Additional file 5: Table S1). 6,881 sites were located on the X and Y chromosomes while 274 sites were located on the autosomal chromosomes (Additional file 5: Table S2). Table 2 reports the number of significant sites per chromosome indicating that gender-related methylation sites are widely spread across the human genome.

**Table 2:** **Number and proportion of significant CpG associations with gender by chromosome**

**Chromosome number**	**Number of sites**	**Proportion of sites**
1	26	0.000558
2	15	0.000435
3	18	0.000720
4	12	0.000592
5	15	0.000621
6	18	0.000494
7	16	0.000536
8	8	0.000385
9	9	0.000924
10	9	0.000372
11	13	0.000454
12	15	0.000615
13	19	0.001560
14	6	0.000400
15	5	0.000330
16	10	0.000457
17	23	0.000828
18	7	0.001189
19	20	0.000785
20	5	0.000485
21	1	0.000238
22	4	0.000470
X	6,669	0.599
Y	212	0.510

We replicated 489 of the identified gender-associated CpGs (*p* < 0.05/468,375) reported by Liu et al. [31], including the previously reported genes like *TLE1* and *TDGF1* [31]. The top 10 autosomal gender-related sites included three hits on the previously reported *TLE1* gene (loci: cg20926353, cg0865632, cg14095100) with *p* values 2.86 × 10^−66^, 2.34 × 10^−57^, and 8.5 × 10^−42^, respectively. TLE1 is a transducin-like enhancer that is important in hematopoiesis and has been involved with acute myeloid leukemia. Interestingly, a gender bias has been observed in the association of TLE1 and different cancers such as acute myeloid leukemia and synovial sarcoma [31].

### Age-related differential methylation

**Figure 2:**
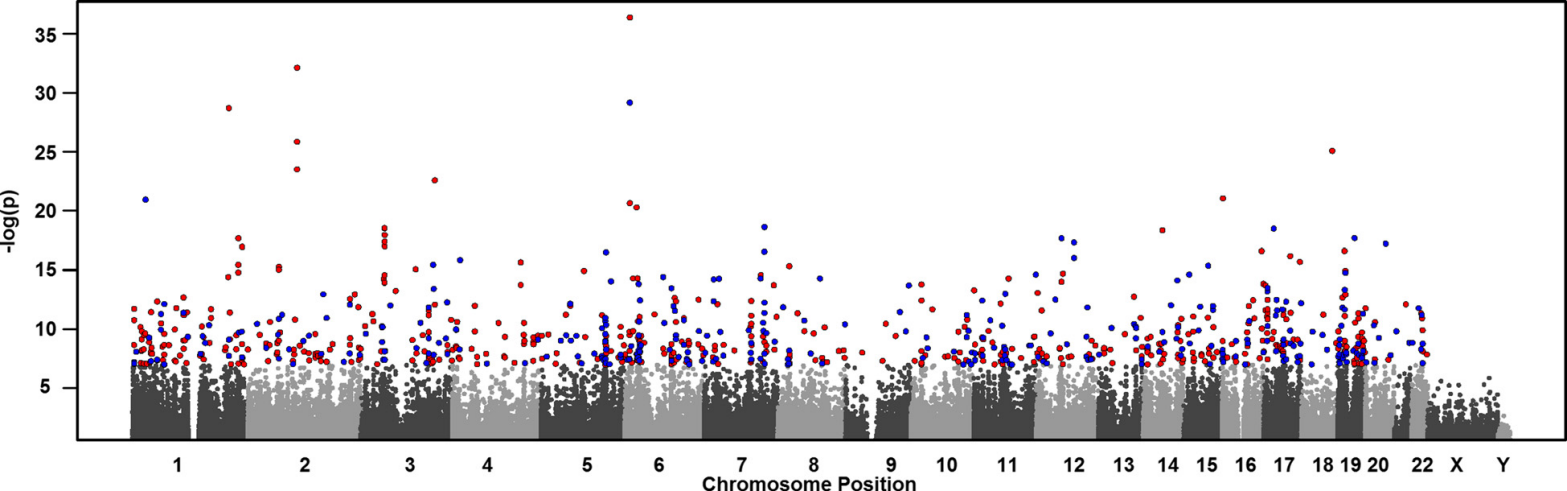
**Manhattan plot for an epigenome-wide association of methylation with age.** Associations with *p* values <1.0675 × 10^−7^ are shown as red dots for sites that are hypermethylated and blue dots for sites that are hypomethylated.

**Figure 3:**
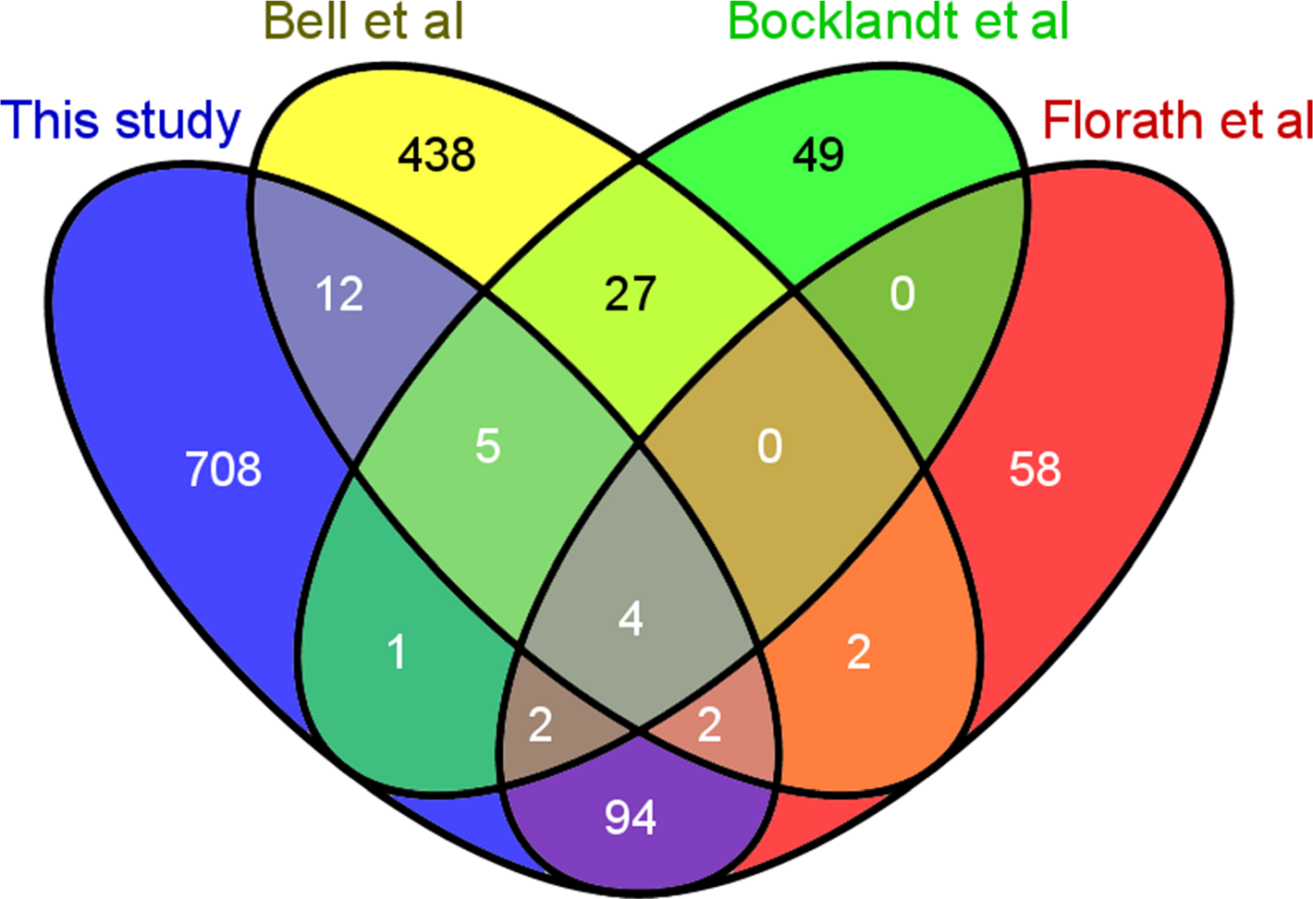
**Venn diagram comparison of age-related differentially methylated loci among different studies (Bell et al.** **[** 26 **]** **, Bocklandt et al.** **[** 40 **]** **, Florath et al.** **[** 30 **]** **).**

Our EWAS between whole blood DNA methylation and age identified 828 significantly associated CpG sites after Bonferroni correction (*p* < 1.07 × 10^−7^). Figure 2 shows the Manhattan plot of the EWAS for age. The most significant age-related differentially methylated site in our study was detected on chromosome 6 in the ELOVL2 gene (ELOVL fatty acid elongase 2) which conforms with another recent study [39] that focused on only monocyte and T-cell lines as opposed to whole blood (as in this paper). A comparison of our findings with three previously published EWAS with age is shown in Figure 3. Considering the overlap between our 468,375 sites and other studies, we replicated 12 out of 88 CpG loci identified by Bocklandt et al. [40], 23 out of 490 CpG loci identified by Bell et al. [26], and 102 out of 162 CpG loci identified by Florath et al. [30], to be highly associated with age. Common associations, found in our study and in the study of Bocklandt et al. [41], were highly significant (i.e. cg09809672 with *p* value 1.17 × 10^−17^, cg21801378 with *p* value 4.5 × 10^−16^, and cg00059225 with *p* value 9.47 × 10^−15^). The top locus reported in [30], cg16867657, ranked first in our list as well with a *p* value of 4.65 × 10^−37^. Also, the top third locus reported in [30], cg21572722, ranked third in our list as well having a *p* value of 7.13 × 10^−30^. The four common age-associated loci among the four studies were cg21801378, cg01820374, cg06291867, and cg04084157 and ranked 38th, 476th, 530th, and 785th, respectively, with *p* values ranging from 4.5 × 10^−16^ to 6.97 × 10^−8^ in our study. A complete table of all age-correlated sites with *p* values below 1 × 10^−7^ (with those replicated in other studies highlighted in yellow) is presented in Additional file 5: Table S3.

**Figure 4:**
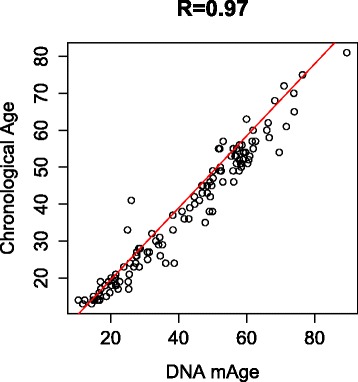
**Correlation plot of DNAm age and chronological age.** DNA mAge was calibrated using the regression model identified from the 353 clock CpGs [12].

Several studies have attempted to use epigenetic data as a predictor of age [34,41]. Bocklandt et al. [40] used a regression model with just two loci that explained a large portion of the variance in age to predict the age of an individual with an accuracy of 5.2 years. More recently, Hovarth et al. [34] developed a tool that uses the methylation status of 353 CpG sites to provide a remarkably accurate age estimate (correlation = 0.97) of the person the cells came from. This tool is a multi-tissue age predictor that is applicable to methylation data from many tissues and cell types. We extracted the 353 discriminative “clock CpGs” from our dataset and computed the age of our study participants using the tool’s identified regression model. To measure the predictive accuracy of the model, we used the Pearson’s correlation coefficient between the DNAm age and chronological age and the median absolute difference between DNAm age and chronological age. These computed measures were correlation = 0.97 and error = 3.7 years which are very close to those previously reported values (correlation = 0.97 and error = 2.9 years). Figure 4 shows the correlation plot that replicates this previous finding in our Arab dataset. Another study used the methylation values of only three CpG sites for chronological age prediction [35]. Applying the multivariate equation of that study to our data as well, we found a 0.85 correlation between predicted age and chronological age with a median absolute error of 7.6 years.

## Discussion

We have combined a number of established techniques to put together a comprehensive pipeline for the preprocessing of epigenetic data and the subsequent analysis for EWASs as indicated in the recent review of appropriate EWAS design [41]. We then used these preprocessed data to perform an EWAS on the phenotypes gender, age, and smoking. Our results have replicated and validated previous findings that were previously identified in the European population. This shows consistency in the effect of these findings in the Arab population under different genetic backgrounds and environmental exposures ensuring validity of the methods applied in the context of epigenome-wide association studies.

The major contribution of this paper is to replicate previous associations of CpG methylation with various phenotypes such as age, gender, and smoking which is important in the context of epigenetic studies. The novelty in this study lies in the application of this type of epigenetic studies on an Arab population. This region of the world has not been explored previously, and we were interested in investigating whether established methods and results are applicable to this interesting population. Our findings encourage expansion of existing projects to accommodate further studies in this underexplored region of the world.

However, we are also aware of some limitations of the present study. For instance, the family structure present in our Qatari dataset (constituting 16 families) displays a high degree of relatedness, which is due to the fact that many of them are family members because of the small size of the Qatari population and cultural traditions. The estimated rate of consanguinity in the Qatari population is 54% [42]. This is quite common in this region of the world but still needs to be accounted for in the analysis of such a dataset. Relatedness can be considered as a confounder in the data and a known cause of Q-Q plot inflation, impacting the association results. Relatedness measures can be expressed in the form of family trees or clusters with some score indicating relative similarity among individuals. An approach of capturing family structure in a condensed form is by computing the principal components (PCs) of the methylation data, although in principle, this can also be performed using the genetic data. The principal components method is known to capture most of the variability in the data in the form of a number of components as described in the Methods section. Visualization of the components against family structure suggests that some of the first ten PCs actually do capture that information (data not shown).

An alternative to computing PCs to account for family structure would be to use a family-based tool such as “lmekin” which implements a linear mixed effects model using a kinship matrix [43]. This tool accepts a family pedigree in the form of a kinship matrix as an additional parameter to the linear mixed effects model and computes the association between each methylation site and a phenotype of interest. When comparing the association results of the linear model described in this paper and the same model parameters applied to lmekin but with the addition of family structure, we found a great similarity in the distribution of *p* values, inflation factors, and even in the robustness in the most significant methylation sites in terms of both ranking and *p* values. Thus, we consider either approach suitable in accounting for a potential relation between methylation and family structure. However, since PCA is designed to capture any confounders that may exist in the data, the extra benefit here is that this might capture any additional hidden variation.

The presence of genetic variation (SNPs) can affect the regulation of methylation and the consequent expression of a phenotype and needs to be addressed when drawing conclusions on methylation-phenotype correlation. Moreover, the presence of SNPs within the probes themselves can result in technical artifacts. The common approach to this problem is to eliminate CpG’s based on the SNP annotation of the Illumina manifest. However, this data is based on tagging SNPs, and it may also be incomplete. Furthermore, it does not fully cover non-Caucasian genomes. In contrast to previous studies, one of the strengths of this paper is the availability of whole-genome sequencing data that is both comprehensive in terms of coverage and specific to our Arab population. This allowed us to correctly remove probes containing polymorphic SNPs, ensuring that all potential confounding genetic variants were eliminated prior to the analysis. In addition, array-based methylation should be validated by another technique. However, since we are reporting associations that have already undergone such validations in studies we cite, repeating such a validation would be beyond the scope of our present paper. Moreover, Illumina has shown high correlation between HumanMethylation 450 K and whole-genome bisulfite sequencing-based methylation [44].

The use of white blood cells as a source for DNA methylation measurement does not take into account the white blood cell distributions that has been shown to have a strong correlation with the methylation signatures [37]. For this reason, we employed a known algorithm for white blood cell count correction before any subsequent analysis. Our EWAS indicates that the influence of both age and gender on methylation is site specific. Both phenotypes display a widespread association throughout the genome and are not limited to specific autosomes. On the autosomes, our data indicated that more sites are highly methylated in females compared to males which is in concordance with a previously published study [31].

Numerous studies observed both age-related hypermethylation [26,27] and hypomethylation [28,29], which we also replicated in our study. Moreover, DNA methylation patterns were shown to increase with age and contribute to age-related diseases such as neurological diseases. Previous studies [25] have analyzed methylation data to test for the association between methylation and age. They clustered the CpGs into classes and evaluated the association of the mean methylation of the CpGs for each class with age. This causes the results to be sensitive to the clustering method depending on the segregation into particular classes, thus capturing only crude associations and possibly missing specific CpGs of interest. Instead, we preferred performing an epigenome-wide association study similar to the ones for the other phenotypes.

Our replication in an Arab population of age prediction with a very high correspondence to what was previously reported [35,40] has particular strength in confirming that these few CpGs do indeed determine or carry information that is age associated independent of population-specific genetics and differing environmental exposures. Although Bocklandt et al. [40] cover a wide range of populations/ethnicities in addition to different cell types and tissues, none of the datasets they used to design their age predictor regression model included datasets from subjects of Arab descent. Therefore, we can be confident that given their algorithm was not trained on similar datasets, yet was able to produce age prediction rates very close to theirs, the same methods can successfully also be applied to non-Caucasian datasets.

Methylation-specific protein binding patterns were found within the aryl hydrocarbon receptor repressor (AHRR) gene with the highest level of changes associated with tobacco smoking. Even maternal smoking during pregnancy had an impact on the epigenome-wide DNA methylation in newborns [23] particularly in the *AHRR* gene. The *AHRR* gene codes for a protein that mediates dioxin toxicity and is involved in regulation of cell growth and differentiation and the modulation of the immune system. The target of AHRR, the aryl hydrocarbon receptor (*AHR*) is a known protein that is a tumor suppressor, mediating detoxification of carcinogenic agents causing tobacco-related lung cancer [45]. Our findings are also in line with other studies [21,22] regarding the *F2RL3* gene, which was first reported to be significantly less methylated in smokers due to the coagulation factor II receptor-like 3 gene and codes for protease-activated receptor 4 (PAR4). It is known to affect platelet activation and other cardiovascular mechanisms such as intimal hyperplasia and inflammation, which are all valid mechanisms for smoking-induced pathology [46].

## Conclusions

In conclusion, we have collected blood samples and phenotype data from an Arab cohort of Qatari origin. The data was processed using established techniques to obtain both methylation measurements and sequencing data. Extensive quality control steps were performed to handle all types of biases ranging from subject-subject variability to blood cellular heterogeneity to hidden confounders. After thorough processing of our dataset, we were able to replicate numerous previously reported methylation sites that were reported to show association with a number of phenotypes namely, age, gender, and smoking.

## Methods

### Methylation data collection

Seven milliliter of whole blood was drawn from the participants and kept in EDTA anticoagulant tubes for DNA extraction. Genomic DNA was extracted with the Qiagen Midi DNA blood extraction kit (Qiagen, spin protocol UK—catalog number 51183) with 2 ml of whole blood including the recommended proteinase K and RNase A digestions. DNA extraction was performed at the Weill Cornell Medical College in Qatar clinical laboratory following the manufacturer instructions. DNA purity and concentration were measured using Qubit 2.0 fluorometer Broad Range kit from Invitrogen (Qubit dsDNA BR Assay kit—catalog numbers Q32850 and Q32853). The DNA samples were stored frozen at −80°C. A 7 ug of the DNA samples were then prepared per sample in a concentration of 70 ng/ul, and the samples were then shipped frozen to Illumina for measurement of DNA methylation on a service for a fee basis, using Illumina’s Infinium HumanMethylation450 BeadChip for interrogating over 485,000 methylation sites. This platform quantifies CpG site methylation using the Illumina DNA bead array technology and DNA bisulfite conversion [47]. The Infinium methylation array uses beads with target-specific probes designed to interrogate CpG sites. The array content included 485,577 assays, out of which 482,421 sites were CpG sites, 3,091 were CpH sites, and 65 were containing SNPs. Based on expert recommendations, CpG site coverage was both comprehensive across complete gene and CpG island regions and biologically significant/informative.

### Whole-genome sequencing data

Data for the SNPs present in each of our subjects was obtained through whole-genome sequencing (WGS) by Illumina using the Hiseq 2500 platform. Paired end sequence reads were obtained with the average depth of coverage of 40×. Sequences were processed by CASAVA (Consensus Assessment of Sequence And VAriation), a propriety bioinformatics pipeline of Illumina, to obtain variant sets. In our study, CASAVA version 1.9 was used which involves aligning of reads to the reference genome, sorting, indexing, realignment, and variant calling. Paired end reads were aligned to the reference human genome of NCBI build 37 using the aligner ELNAD v2 (Efficient Large-Scale Alignment of Nucleotide Database) in the CASAVA pipeline. Variant calling utilizes a probabilistic algorithm to call the genomic consensus sequence and compares it to the reference sequence in order to identify homozygous or heterozygous SNPs. For each of the variants called, CASAVA also provides quality measures. The SNPs were filtered based on the quality score provided, to retain variants with error probability less than 0.01.

### Preprocessing pipeline

Confirmation checks to ensure our sample integrity first included verifying Mendelian inheritance by looking at the whole-genome sequencing data. Trios were checked for Mendelian violations among their SNPs, and the average acceptable percentage of violations did not exceed 10% in all trios. Some initial filtering was performed on the Illumina-provided methylation data. The percentage of detected sites was on average 99.5% (with a *p* value <0.01). No samples were excluded based on the number of detected sites. The overall signal intensity and the distribution of M values of the samples were then inspected. The M value is simply a logit transform of the B-value, which are both used interchangeably [48]. No samples had low overall signal intensities or abnormal methylation profiles.

Initially, there were 485,577 probes, and after filtering, 482,421 CpG probes remained (3,091 were CpH probes and 65 probes tag SNPs from Illumina manifest). 11,135 CpG probes were on the X chromosome, and 416 were on the Y chromosome. The X and Y chromosome probes were only filtered when studying differential methylation for smoking and age, but not gender, to remove potential bias from the possibility of different proportions between females and males. Then, the detection *p* values of the methylation sites were inspected. They reflected the strength of DNA hybridization over the background (comparing the CpG intensity with the intensities of negative control probes). A total of 2,495 probes had a detection *p* value greater than 0.01 in 5% of the samples and were excluded. After all the filtering, 468,375 methylation sites remained under consideration. Genetic variants or SNPs in probes or CpG sites can interfere with methylation readouts by affecting probe binding. Therefore, we set all methylation data to missing values whenever a genetic variant existed within the region of +/−110 base pairs of the CpG, based on our whole-genome sequencing data. These accounted for about 0.5% of the methylation sites that were excluded.

There are different sort of biases in the Infinium HumanMethylation450 BeadChip assay such as color channel bias and probe type bias. Numerous methods exist to handle different kinds of biases, and there is an established best practice to handle such biases [36]. The Lumi:QN + BMIQ pipeline was applied to our dataset as prior processing. This pipeline was implemented and applied to our dataset using the bioconductor “lumi” package [49]. The Infinium HumanMethylation450 BeadChip assay includes Infinium I and Infinium II study designs. In the former design, two different probes (corresponding to the methylated and unmethylated alleles) located on two different bead types and the methylated and unmethylated signals are generated in the same color channel. In the latter design, a single base extension from the 3′ end of the probe sequence (which is one base upstream of the query base) will result in either a red or green signal depending on whether the query site was unmethylated or methylated. Illumina uses two colors to label the final extended base following the hybridization of methylated or unmethylated probes. As a result, some of the CpG sites are measured in the red channel (final extended bases are A or T), whereas others are measured in the green channel (final extended bases are C or G). The methylated and unmethylated probes of the same CpG site have the same color. Due to the difference in labeling efficiency and scanning properties of two color channels, the intensities measured in two color channels might be imbalanced. The basic idea of color balance adjustment is to treat it as the normalization between two color channels. Because the two color channels have a different number of probes that do not match each other, the regular quantile normalization cannot be directly applied, instead the smooth quantile normalization method is used [49].

The total CpG methylation can differ significantly from sample to sample in different conditions. Quantile normalization (QN) is used to reduce between sample variations and centers the signal between arrays (correcting for influence of position on the slide). However, directly applying the normalization methods to the methylation data, like M value or beta value, is inappropriate. Instead, normalization is performed at the probe level, ie. the intensities of methylated and unmethylated probes are normalized instead of their summarized level.

The probe-type bias is not sufficiently reduced by just QN, and beta mixture quantile dilation (BMIQ) normalization is needed [50]. The bias between probe types 1 and 2 is optimally reduced given that type 1 probes are more likely to map to CpG islands than type 2 (the proportions of methylated and unmethylated probes vary between the two types) and that the density distributions of the two types should be matched, especially at the methylated/unmethylated extremes. The expectation maximization (EM) algorithm is used in BMIQ normalization with three states in the beta mixture model.

### Adjusting for cellular heterogeneity

The Houseman software we used to adjust for cell type is described in [37]. Cell distribution might differ by disease status, thus cell heterogeneity may act as a confounder when investigating DNA methylation differences. Quantification of overall lymphocyte composition can only be done using methods based on flow cytometry. This requires large volumes of fresh blood and labor-intensive antibody tagging. Given that the DNA methylation signature is highly correlated with the leukocyte distribution, the methylSpectrum method [37] performs a deconvolution algorithm similar to quadratic programming and regression calibration to investigate association with a disease. Using external validation data, the model is calibrated and the bias is corrected for.

The Houseman method performs a deconvolution of DNA methylation array data into source contributions from distinct cell types by determining the composition of white blood cells from DNA methylation array data assayed in whole blood. The approach depends on the DNA methylation signature of each of the principal immune components of whole blood that includes B cells, granulocytes, monocytes, NK cells, and T cells. The methylation signature is considered a high-dimensional multivariate surrogate for the distribution of white blood cells, which can be used in predicting disease states. Because the DNA methylation signature is thought to be highly correlated to the leukocyte distribution, this fits into the framework of measurement error models where using a noisy surrogate marker to test the association with a disease results in biased estimates, unless validation data can be obtained to calibrate the model and correct the bias.

### Adjusting for potential confounders

Many differences between subjects can exist due to varying life styles, diet, or medication. When performing an EWAS, the major sources of variation in the methylation data must be captured and regressed out in association analyses. Principal component analysis (PCA) is used to capture the unmeasured sources of variation in methylation data using the MethylPCA tool [39]. The data is first reduced in size by combining methylation data from neighboring sites. The measured and computed covariates (including age, gender, batch, and white blood cell composition) were regressed out prior to PCA. The computed PCs were considered additional covariates that were supplied to the multiple linear regression when testing for the association between phenotype and each methylation site.

### Statistical analysis

Association tests were performed to identify sites where methylation varies with a given phenotype. The influence of any phenotype on methylation was done by identifying the differentiated genes based on the linear model “lm” in R. There were a large number of covariates for each of the association studies, including age, gender, BMI, batch, six white blood cell coefficients, and ten PCs. To avoid over-fitting by regressing out too many parameters in the different models, we tested each individual covariate against the phenotype of interest and using the R “anova” command and determined whether the addition of a particular covariate was significant. We only added covariates that resulted in a better fit to the model with a *p* value <0.05. The covariates that were to be incorporated into the different models were incrementally and independently selected for each association study. For the gender association study, the best linear model included the CpG sites and age, BMI, only two of the white blood cell coefficients (NK cells and B cells), and only three of the PCs (PC7, PC6, and PC9) as covariates. For the age association study, the best linear model included the CpG sites and BMI, gender, only two white blood cell coefficients (CD8^+^T cells and monocytes), and only five PCs (PC4, PC3, PC2, PC10, and PC5) as covariates. For the smoking association study, the best linear model included the CpG sites and the gender, BMI, age, only two of the cell coefficients (monocytes and granulocytes), only the first PC (PC1), and batch as covariates. We applied different models such as CpG ~ age + gender + BMI, CpG ~ age + gender + BMI + cell coefficients, and CpG ~ age + gender + BMI + cell coefficients + PCs and monitored the changes in inflation in the genome-wide association studies. This produced a set of *p* values that can be represented as Q-Q plots and Manhattan plots. This is followed by multiple testing adjustments, and mean levels of methylations were compared across phenotype categories while adjusting for the known and computed confounders in the form of PCs. The IlluminaHumanMethylation450k.db annotation package was used for the annotation of CpGs so that most of the differentially methylated CpG sites were mapped to some gene name.

### Declarations

#### Ethics approval and consent to participate

The study was conducted with prior Institutional Review Board approval of Weill Cornell Medical College in Qatar and in concordance with the Helsinki declaration of ethical principles for medical research involving human subjects (ethical approval numbers 2012–003 and 2012–0025). Subjects provided written informed consent for the collection and subsequent analysis of samples.

## Additional files

Additional file 1: Figure S1. DNA methylation for the 123 samples presented as boxplots. The circles represent outliers, and the red and green boxes represent the two color channels showing the effect of the quality control on the data **a)** before preprocessing, **b)** after color bias adjustment, and **c)** after quantile normalization. Additional file 2: Figure S2. Example of the methylation profile of an arbitrary subject showing the effect of BMIQ normalization **a)** before and **b)** after BMIQ normalization. The extra peak in **a)** is due to the probe bias. Additional file 3: Figure S3. Calibrated white blood cell distribution plots. Additional file 4: Figure S4. Scree plot showing the proportion of variance accounted for by each individual principal component for the smoking phenotype. Additional file 5: Table S1. List of genome-wide gender-related differentially methylated CpG sites. **Table S2.** List of gender-related differentially methylated CpG sites occurring on autosomes only. **Table S3.** List of genome-wide age-related differentially methylated CpG sites. Highlighted entries are replicated in at least one other study.
