# Supplementary material for: Association of DNA methylation with age, gender, and smoking in an Arab population
Source: Clin Epigenetics. 2015 Jan 22;7(1):6. doi: 10.1186/s13148-014-0040-6 (PMC4320840; doi:10.1186/s13148-014-0040-6)
Supplement: Additional file 1: Figure S1. — DNA methylation for the 123 samples presented as boxplots. The circles represent outliers, and the red and green boxes represent the two color channels showing the effect of the quality control on the data a) before preprocessing, b) after color bias adjustment, and c) after quantile normalization. [file 13148_2014_40_MOESM1_ESM.pdf]

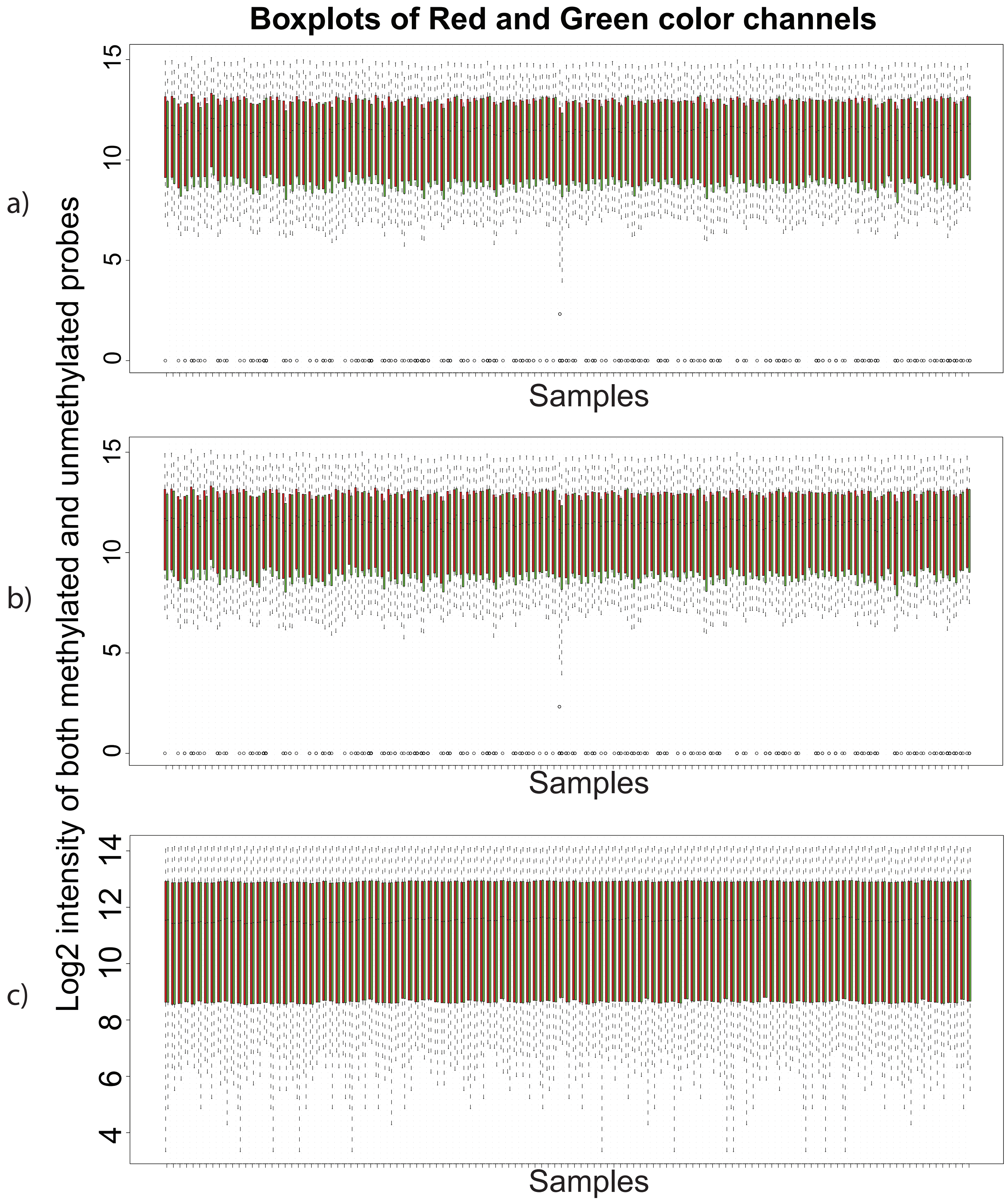
Boxplots of Red and Green color channels
a)
b)
c)
Log2 intensity of both methylated and unmethylated probes
Samples
Samples
Samples
0
5
10
15
4
6
8
10
12
14
