# Supplementary figures and images for: Association of DNA methylation with age, gender, and smoking in an Arab population
Source: Clin Epigenetics. 2015 Jan 22;7(1):6. doi: 10.1186/s13148-014-0040-6 (PMC4320840; doi:10.1186/s13148-014-0040-6)

**a) Before BMIQ Normalization**

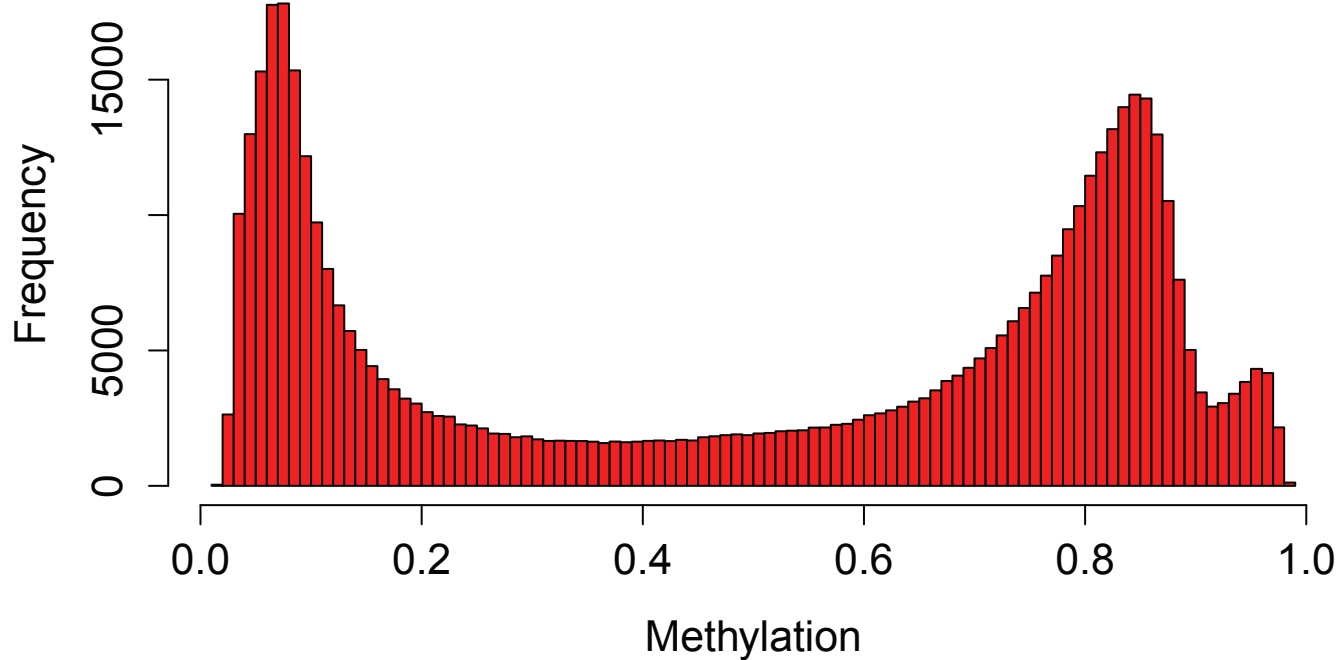

**b) After BMIQ Normalization**

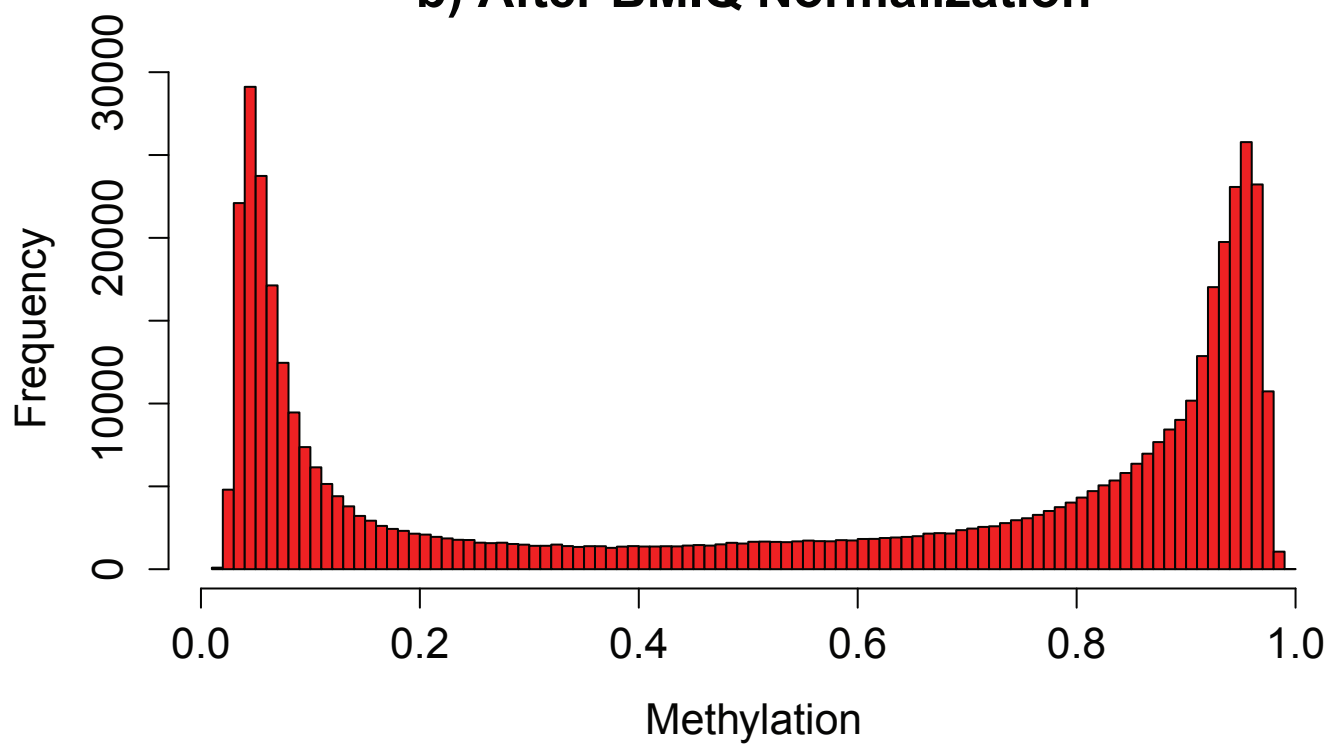

Supplement: Additional file 2: Figure S2. — Example of the methylation profile of an arbitrary subject showing the effect of BMIQ normalization a) before and b) after BMIQ normalization. The extra peak in a) is due to the probe bias. [file 13148_2014_40_MOESM2_ESM.pdf]

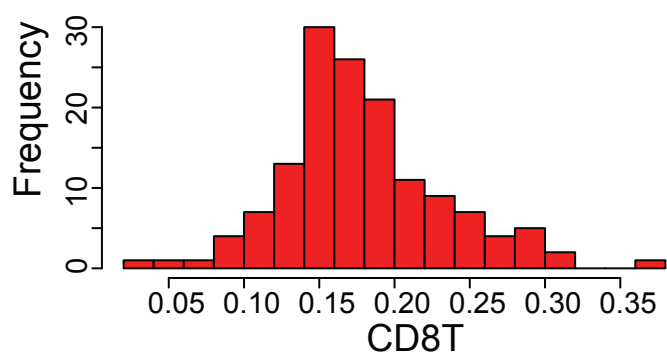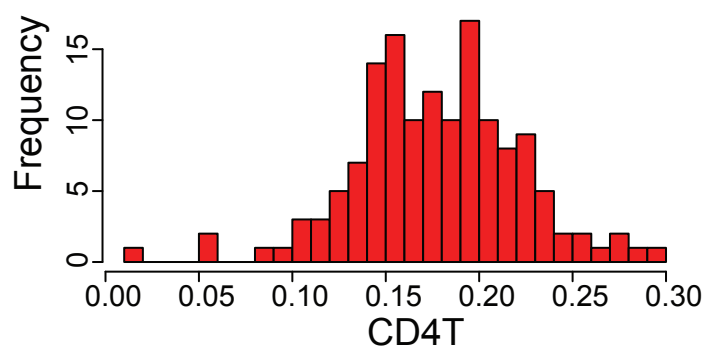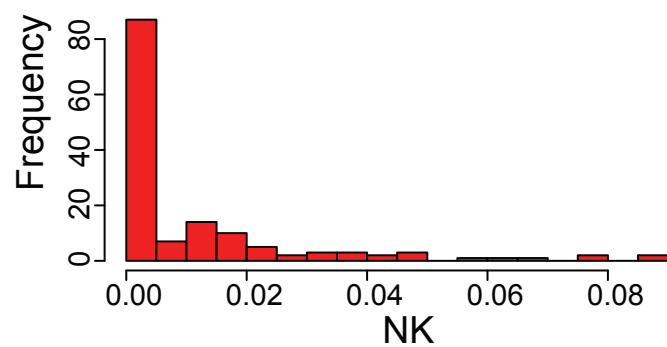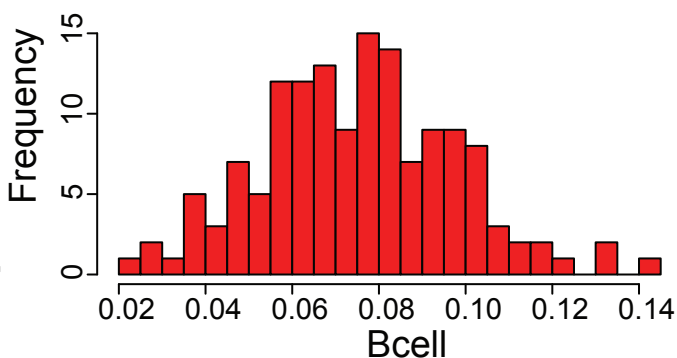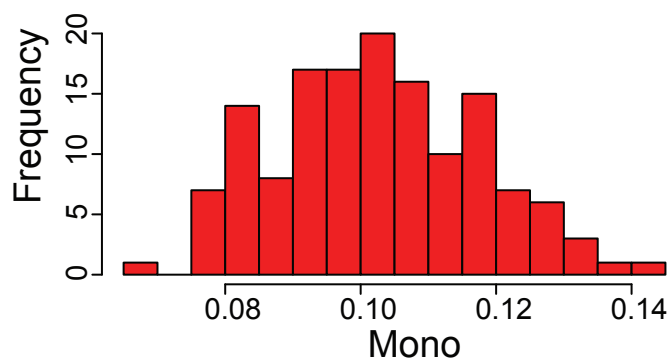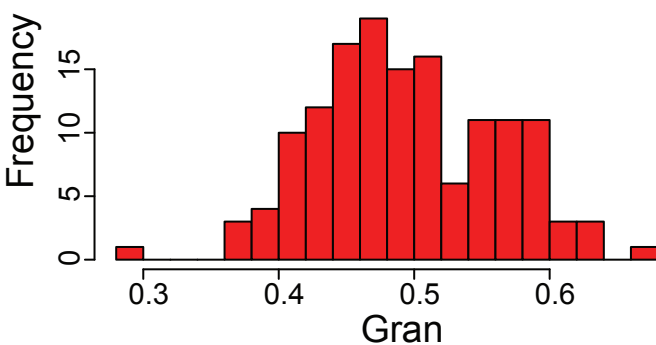

Supplement: Additional file 3: Figure S3. — Calibrated white blood cell distribution plots. [file 13148_2014_40_MOESM3_ESM.pdf]

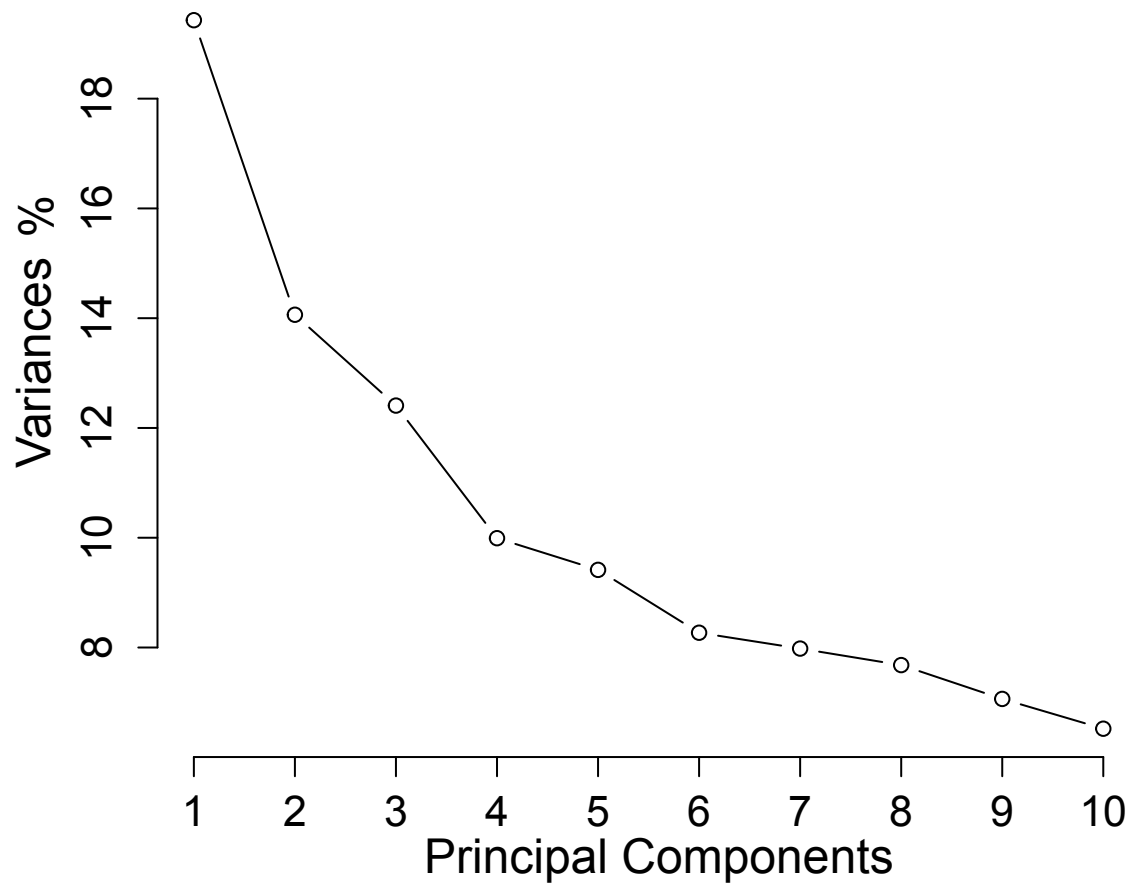

Supplement: Additional file 4: Figure S4. — Scree plot showing the proportion of variance accounted for by each individual principal component for the smoking phenotype. [file 13148_2014_40_MOESM4_ESM.pdf]
